# Balloon endoscopy-assisted endoscopic retrograde cholangiopancreatography for hepatolithiasis in patients with hepaticojejunostomy

**DOI:** 10.1007/s00464-024-10738-6

**Published:** 2024-03-07

**Authors:** Ryunosuke Hakuta, Tatsuya Sato, Yousuke Nakai, Hirofumi Kogure, Hiroto Nishio, Kouhei Kurihara, Shuichi Tange, Rintaro Fukuda, Shinya Takaoka, Yukari Suzuki, Hiroki Oyama, Sachiko Kanai, Kensaku Noguchi, Tatsunori Suzuki, Kazunaga Ishigaki, Tomotaka Saito, Tsuyoshi Hamada, Naminatsu Takahara, Mitsuhiro Fujishiro

**Affiliations:** 1 Department of Gastroenterology, Graduate School of Medicine, The University of Tokyo, Tokyo, Japan; 2 Department of Endoscopy and Endoscopic Surgery, The University of Tokyo, Tokyo, Japan; 3 Division of Gastroenterology and Hepatology, Department of Medicine, Nihon University School of Medicine, Tokyo, Japan; 4 Department of Chemotherapy, Graduate School of Medicine, The University of Tokyo, Tokyo, Japan

**Keywords:** Endoscopic retrograde cholangiopancreatography, Enteroscopy, Gallstones, Hepaticojejunostomy

## Abstract

**Background and aim:**

Balloon endoscopy-assisted endoscopic retrograde cholangiopancreatography (BE-ERCP) is an emerging procedure for pancreatobiliary diseases in patients with surgically altered anatomy. However, data on BE-ERCP for hepatolithiasis after hepaticojejunostomy (HJS) are still limited.

**Methods:**

Stone removal success, adverse events and recurrence were retrospectively studied in consecutive patients who underwent BE-ERCP for hepatolithiasis after HJS between January 2011 and October 2022. Subgroup analysis was performed to compare clinical outcomes between patients who had undergone HJS over 10 years before (past HJS group) and within 10 years (recent HJS group).

**Results:**

A total of 131 patients were included; 39% had undergone HJS for malignancy and 32% for congenital biliary dilation. Scope insertion and complete stone removal were successful in 89% and 73%, respectively. Early adverse events were observed in 9.9%. Four patients (3.1%) developed gastrointestinal perforation but could be managed conservatively. Hepatolithiasis recurrence rate was 17%, 20% and 31% in 1-year, 3-year, and 5-year after complete stone removal. The past HJS group was the only risk factor for failed stone removal (odds ratio 10.4, 95% confidence interval 2.99–36.5) in the multivariable analysis. Failed scope insertion (20%) and failed guidewire or device insertion to the bile duct (22%) were two major reasons for failed stone removal in the past HJS group.

**Conclusions:**

BE-ERCP for hepatolithiasis was effective and safe in cases with HJS but the complete stone removal rate was low in the past HJS group. Recurrent hepatolithiasis was common and careful follow up study is needed even after complete stone removal.

**Supplementary Information:**

The online version contains supplementary material available at 10.1007/s00464-024-10738-6.

Hepatolithiasis, stones in the intrahepatic bile ducts, can cause cholangitis, liver abscess, or obstructive jaundice, and guidelines recommend treatment including endoscopic retrograde cholangiopancreatography (ERCP), percutaneous transhepatic biliary drainage (PTBD), or surgery [1, 2] PTBD was widely performed as a first-line non-surgical treatment for hepatolithiasis [3, 4] but there was a concern in impairing activity of daily life and risk of early adverse events including bleeding [5]. A recent study suggested comparative effectiveness of ERCP and PTBD [6], and ERCP is now a treatment option for hepatolithiasis in clinical practice.

ERCP in patients with surgically altered anatomy (SAA), however, is technically challenging. Balloon endoscopy-assisted ERCP (BE-ERCP) is an emerging procedure for this population and previous studies reported its effectiveness and safety [7–9]. However, the clinical evidence is still limited to date regarding feasibility of BE-ERCP for hepatolithiasis in patients with SAA [5, 10]. Considering a low clearance rate of hepatolithiasis via ERCP even in patients with normal anatomy [6], effectiveness and safety of BE-ERCP needs to be validated in this cohort. Furthermore, clinical outcomes of BE-ERCP could be different between patients who underwent surgery in their childhood and adulthood due to different etiology (congenital biliary dilation or malignancy), as well as gastrointestinal reconstruction (Roux-en-Y or Billroth-II).

Therefore, we conducted the current study to elucidate clinical outcomes and risk factors for failed complete stone removal via BE-ERCP for hepatolithiasis after hepaticojejunostomy (HJS).

## Methods

### Study design

The current study was a single-center retrospective study to evaluate the effectiveness and safety of BE-ERCP for hepatolithiasis after HJS. This study was conducted according to the guidelines in the Declaration of Helsinki and was approved by the ethics committee of the University of Tokyo. Written informed consent for procedure was obtained from each patient before the procedure. Consent for use of data was obtained on a basis of the opt-out consent.

### Patients

Consecutive patients who underwent BE-ERCP for hepatolithiasis after HJS during the study period were identified through our ERCP database. Hepatolithiasis were defined as stones in the intrahepatic bile duct. Patients with choledochojejunostomy were not included in the analysis. Exclusion criteria were as follows: (1) patients who underwent transpapillary procedure, (2) patients who underwent BE-ERCP for benign biliary stricture alone (without hepatolithiasis), and (3) patients with malignant biliary obstruction. For patients who underwent repeated BE-ERCP for hepatolithiasis during the study period, only the first session was included for the analyses. The primary outcome was a rate of complete stone removal via BE-ERCP. The secondary outcomes included a rate of final complete stone removal, early adverse events associated with BE-ERCP, and a cumulative incidence of recurrent hepatolithiasis after complete stone removal. For the evaluation of the recurrent hepatolithiasis, patients with failed stone removal via BE-ERCP, patients who underwent subsequent biliary stent placement for concomitant biliary stricture, patients without follow up > 30 days were excluded from analyses.

### Endoscopic procedures

A short type double-balloon endoscope (EC-450BI5/EI-530B with a 2.8-mm-wide working channel or EI-580BT with a 3.2-mm-wide working channel; Fujifilm, Tokyo, Japan) was utilized for BE-ERCP. EI-580BT was consecutively used from July 2015 [11]. Most standard ERCP devices are applicable to the scope other than mother-baby cholangioscopy due to the small working channel. All patients were admitted for BE-ERCP procedures. The details of BE-ERCP procedures in our institution were reported elsewhere [12, 13].

The algorithm of removal of hepatolithiasis is shown in Supplementary Table 1. Balloon dilation was performed in cases with concomitant biliary stricture. A diameter of balloon was selected not to exceed a diameter of the bile duct just above the stricture. Basket or balloon catheter was used for removal of hepatolithiasis. Endoscopic mechanical lithotripsy (EML) was performed if stones were larger than the diameter of HJS. For patients with failed stone fragmentation via EML, electrohydraulic lithotripsy (EHL) under direct cholangioscopy [14] or extracorporeal shock wave lithotripsy (ESWL) after placement of endoscopic nasobiliary drainage were performed. For patients with failed complete stone removal via BE-ERCP, PTBD, endoscopic ultrasonography-guided hepaticogastrostomy (EUS-HGS), surgery, or conservative treatment was selected according to each patient’s condition [15]. Generally, patients with hepatolithiasis in the right lobe underwent PTBD and those in the left lobe underwent EUS-HGS. Patients with persistent biliary stricture after balloon dilation underwent plastic or metal stent placement across the stricture after stone removal [16].

### Definitions of outcome variables and follow-up strategy

Early adverse events and their severities were defined according to the lexicon by American Society of Gastrointestinal Endoscopy [17]. The severity of early adverse event was graded as follows: mild, requiring an unplanned prolongation of hospital stay for ≤ 3 days; moderate, requiring an unplanned prolongation of hospital stay for 4 to 10 days, endoscopy, interventional radiology, or admission to the intensive care unit for 1 night; and severe, requiring an unplanned prolongation of hospital stay for > 10 days, admission to the intensive care unit for > 1 night, or surgical intervention. Complete stone removal was defined as absence of bile duct stones via balloon occluded cholangiography during BE-ERCP or computed tomography (CT).

After successful stone removal, patients were followed regularly in the outpatient clinic at least every three months. In each visit, laboratory test including liver enzymes and inflammatory markers were evaluated and imaging studies with abdominal ultrasound, CT, or magnetic resonance cholangiopancreatography were performed every six months.

### Statistical analysis

Categorical variables were compared using the chi-square test or Fisher’s exact test, as appropriate. Continuous variables were compared using the Wilcoxon rank-sum test.

In subgroup analyses, patients were divided into two groups by a period from surgery of HJS to BE-ERCP. Briefly, patients who had undergone the index surgery being over 10 years prior to the index BE-ERCP were categorized in the past HJS group, compared with who underwent surgery within 10 years were in the recent HJS group. Baseline characteristics and clinical outcomes were compared between the past and the recent HJS groups.

Uni- and multi-variable logistic regression models were used to estimate factors associated with failed stone removal via BE-ERCP. The multivariable model included variables with their *P* value < 0.10 in the univariable model. Cumulative incidences of recurrent hepatolithiasis were estimated using the Kaplan–Meier method and compared using the log-rank test. The lost follow-up or death were dealt as censored.

For all analyses, a two-sided *P* value < 0.05 was used to denote statistical significance. All statistical analyses were performed using the EZR software (Saitama Medical Center, Jichi Medical University, Saitama, Japan), which is a graphical user interface for the R software (The R Foundation for Statistical Computing, Vienna, Austria, version 3.4.1) [18].

## Results

### Patient characteristics

**Fig. 1:**
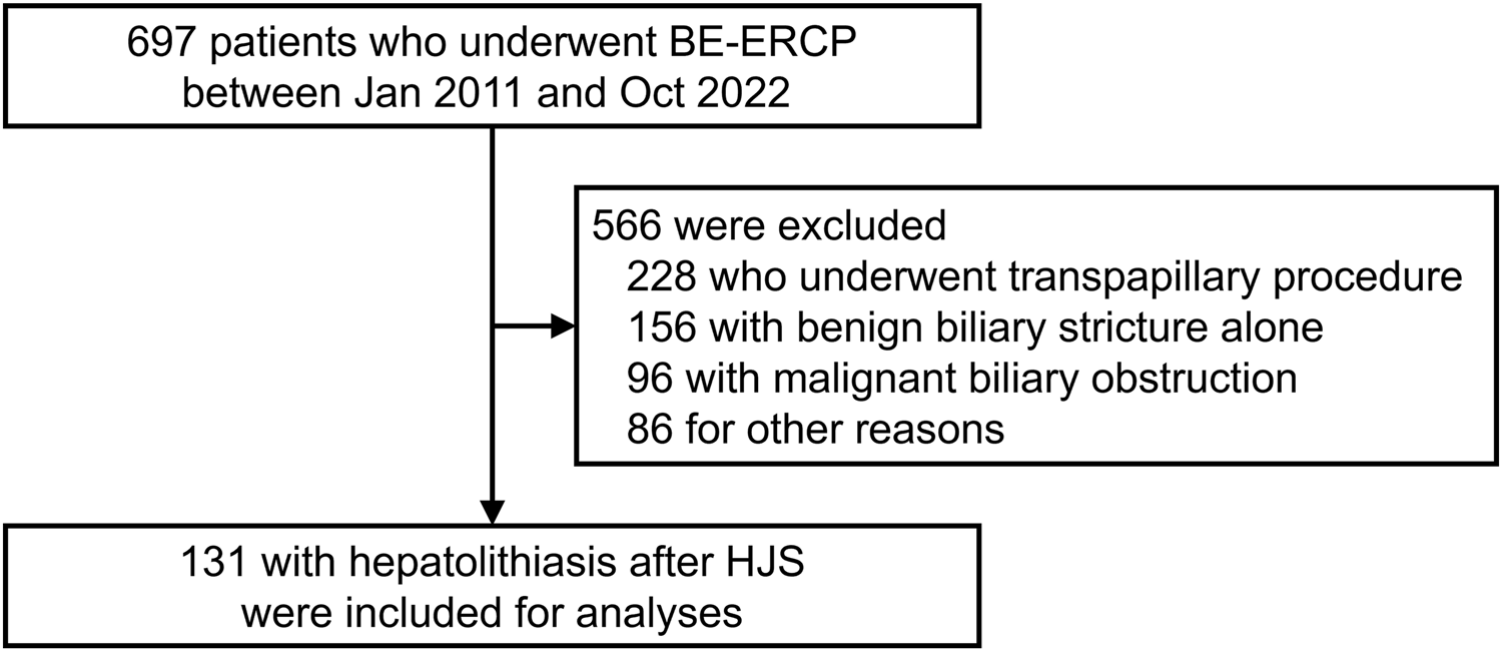
Flowchart of patients undergoing BE-ERCP for hepatolithiasis with HJS. *BE-ERCP* balloon endoscopy-assisted endoscopic retrograde cholangiopancreatography, *HJS* hepaticojejunostomy

Between January 2011 and October 2022, among 697 patients who underwent BE-ERCP in our institution (Fig. 1), 131 patients underwent BE-ERCP for hepatolithiasis after HJS. Baseline characteristics are shown in Table 1. The median age was 63 years, and primary disease was malignancy in 39% and congenital biliary dilation in 32%. As for gastrointestinal reconstruction, 71% of patients had Roux-en-Y reconstruction and 27% had Billroth-II reconstruction. Median maximum stone diameter was 9 mm, and 61% of patients had multiple stones (the number of stones was two or more).

**Table 1:** Baseline characteristics of patients after HJS who underwent BE-ERCP for hepatolithiasis

Characteristic^a^	Total (*n* = 131)
Age, years	63 (48–74)
Gender, male	68 (52%)
Primary disease	
Malignancy	51 (39%)
Pancreas	27 (21%)
Biliary tract	19 (15%)
Other	5 (3.8%)
Congenital biliary dilation	42 (32%)
Congenital biliary atresia	6 (4.6%)
Other benign diseases	32 (24%)
Type or surgery	
Pancreaticoduodenectomy	56 (43%)
Extrahepatic bile duct resection	63 (48%)
Other	12 (9.2%)
GI reconstruction	
Roux-en-Y	93 (71%)
Billroth-II	36 (27%)
Braun anastomosis	27 (21%)
Other	2 (1.5%)
Biliary stricture	74 (57%)
Maximum stone diameter, mm	9 (6–14)
Multiple stone^b^	80 (61%)
Stone location, hilar^c^	67 (51%)
Stone distribution, bilateral	25 (19%)
Time from surgery to BE-ERCP, years	8 (3–20)

*BE-ERCP* balloon endoscopy-assisted endoscopic retrograde cholangiopancreatography, *GI* gastrointestinal, *HJS* hepaticojejunostomy

^a^Data are expressed as number (percentage) of patients within a given group or as median (interquartile range)

^b^The number of stone two or more was defined as multiple stone

^c^Hilar was defined as common, left, or right hepatic duct

### Clinical outcomes of BE-ERCP

Table 2 shows the details of endoscopic procedures. The rate of successful scope insertion to HJS was 89% and the median time for scope insertion was 19 min. The rate of lithotripsy use was 28%; EML in 18%, ESWL in 9.2%, and EHL in 6.1%. Complete stone removal via BE-ERCP was achieved in 73% in a median of 1 (interquartile range 1–2) BE-ERCP session. The reasons for failed stone removal were failed scope insertion in 11% and failed guidewire or device insertion to an intended bile duct in 11%. As a rescue procedure after failed stone removal via BE-ERCP, PTBD, EUS-HGS, and surgery were performed in 15%, 9.9% and 0.8%, respectively. Conservative management without interventions was selected in 3.8%. As a result, final complete stone removal was achieved in 92% of patients. The uni- and multi-variable analyses of risk factors for failed stone removal via BE-ERCP are shown in Table 3. The multivariable model showed the past HJS group as the only risk factor for failed stone removal via BE-ERCP (odds ratio 10.4, 95% confidence interval 2.99–36.5).

**Table 2:** The details of endoscopic procedures for removal of hepatolithiasis in patients with HJS

Procedure^a^	Total (*n* = 131)
Type of scope	
EC-450BI5 or EI-530B	43 (33%)
EI-580BT	88 (67%)
Successful scope insertion to HJS	116 (89%)
Scope insertion time, minutes	19 (10–33)
Procedure time in the first session, minutes	74 (48–104)
Lithotripsy	36 (28%)
EML	24 (18%)
ESWL	12 (9.2%)
EHL	8 (6.1%)
Number of sessions	1 (1–2)
Complete stone removal via BE-ERCP	95 (73%)
Reason for failed stone removal via BE-ERCP	
Failed scope insertion	15 (11%)
Failed guidewire or device insertion to bile duct	15 (11%)
Other	6 (4.6%)
Treatment after failed endoscopic stone removal	
PTBD	19 (15%)^b^
EUS-HGS	13 (9.9%)^b^
Conservative	5 (3.8%)
Surgery	1 (0.8%)
Final complete stone removal	121 (92%)

*BE-ERCP* balloon endoscopy-assisted endoscopic retrograde cholangiopancreatography, *EHL* endoscopic hydraulic lithotripsy, *EML* endoscopic mechanical lithotripsy, *ESWL* extracorporeal shockwave lithotripsy, *EUS-HGS* endoscopic ultrasonography-guided hepaticogastrostomy, *HJS* hepaticojejunostomy, *PTBD* percutaneous transhepatic biliary drainage

^a^Data are expressed as number (percentage) of patients within a given group or as median (interquartile range)

^b^Two patients underwent both PTBD and EUS-HGS

**Table 3:** Uni- and multi-variable logistic regression analyses to assess factors associated with failed stone removal via BE-ERCP

Subgroup	No.	FSR, *n* (%)	OR (95% CI) for FSR			
			Univariable	*P* value	Multivariable^a^	*P* value
Group^b^						
Recent HJS	67	4 (6.0%)	1 (referent)		1 (referent)	
Past HJS	64	32 (50%)	15.7 (5.12–48.4)	< 0.001	10.4 (2.99–36.5)	< 0.001
Age						
< 65 years	68	25 (37%)	1 (referent)		1 (referent)	
≥ 65 years	63	11 (18%)	0.36 (0.16–0.82)	0.02	1.23 (0.35–4.35)	0.74
Gender						
Female	63	21 (33%)	1 (referent)			
Male	68	15 (22%)	0.57 (0.26–1.23)	0.15		
Primary disease						
Other	32	6 (19%)	1 (referent)		1 (referent)	
Congenital biliary dilation or atresia	48	23 (48%)	3.99 (1.39–11.4)	0.01	1.67 (0.36–7.64)	0.51
Malignancy	51	7 (14%)	0.69 (0.21–2.27)	0.54	1.57 (0.35–7.00)	0.55
Type of surgery						
Other	12	4 (33%)	1 (referent)		1 (referent)	
Pancreaticoduodenectomy	56	6 (11%)	0.24 (0.06–1.04)	0.057	0.34 (0.04–2.79)	0.31
Bile duct resection	63	26 (41%)	1.41 (0.38–5.16)	0.61	0.86 (0.16–4.68)	0.86
GI reconstruction/Roux-en-Y						
No	38	3 (7.9%)	1 (referent)		1 (referent)	
Yes	93	33 (36%)	6.42 (1.83–22.5)	0.004	0.80 (0.12–5.55)	0.82
Type of scope						
EC-450BI5 or EI-530B	43	12 (28%)	1 (referent)			
EI-580BT	88	24 (27%)	0.97 (0.43–2.19)	0.94		
Stone diameter > 10 mm						
No	67	21 (31%)	1 (referent)			
Yes	64	15 (23%)	0.67 (0.31–1.46)	0.31		
Multiple stone^c^						
No	51	15 (29%)	1 (referent)			
Yes	80	21 (26%)	1.63 (0.45–5.94)	0.46		
Stone location						
Hilar^d^	67	11 (16%)	1 (referent)		1 (referent)	
Peripheral	64	25 (39%)	3.26 (1.44–7.40)	0.005	1.54 (0.56–4.23)	0.82
Stone distribution						
Unilateral	106	31 (29%)	1 (referent)			
Bilateral	25	5 (20%)	0.61 (0.21–1.76)	0.36		
Biliary stricture						
No	57	23 (40%)	1 (referent)		1 (referent)	
Yes	74	13 (18%)	0.32 (0.14–0.70)	0.005	0.50 (0.19–1.31)	0.16

*BE-ERCP* balloon endoscopy-assisted endoscopic retrograde cholangiopancreatography, *CI* confidence interval, *FSR* failed stone removal, *GI* gastrointestinal, *HJS* hepaticojejunostomy, *OR* odds ratio

^a^Variables with *P* value < 0.10 in the univariable model were entered into the

^b^Patients were divided into the past and recent HJS groups regarding time from surgery to BE-ERCP (> 10 years or ≤ 10 years)

^c^The number of stone two or more was defined as multiple stone

^d^Hilar was defined as common, left, or right hepatic duct

Early adverse events were observed in 9.9% (Table 4). Mild cholangitis was the most frequent adverse event (5.3%). Four patients (3.1%) developed gastrointestinal perforation, but all of them could be managed conservatively; severity was mild in 3 and moderate in 1. Among 19 patients who underwent PTBD, early adverse events were developed in 26%.

**Table 4:** Early adverse events associated with BE-ERCP for hepatolithiasis in patients with HJS

Early adverse events^a^	Total (*n* = 131)
Total	13 (9.9%)
Cholangitis	7 (5.3%)
Mild/moderate/severe	7/0/0 (5.3%/0%/0%)
Gastrointestinal perforation	4 (3.1%)
Mild/moderate/severe	3/1/0 (2.3%/0.8%/0%)
Bleeding	1 (0.8%)
Mild/moderate/severe	1/0/0 (0.8%/0%/0%)
Bile duct injury	1 (0.8%)
Mild/moderate/severe	1/0/0 (0.8%/0%/0%)

^a^Data are expressed as number (percentage) of patients

*BE-ERCP* balloon endoscopy-assisted endoscopic retrograde cholangiopancreatography, *HJS* hepaticojejunostomy

**Fig. 2:**
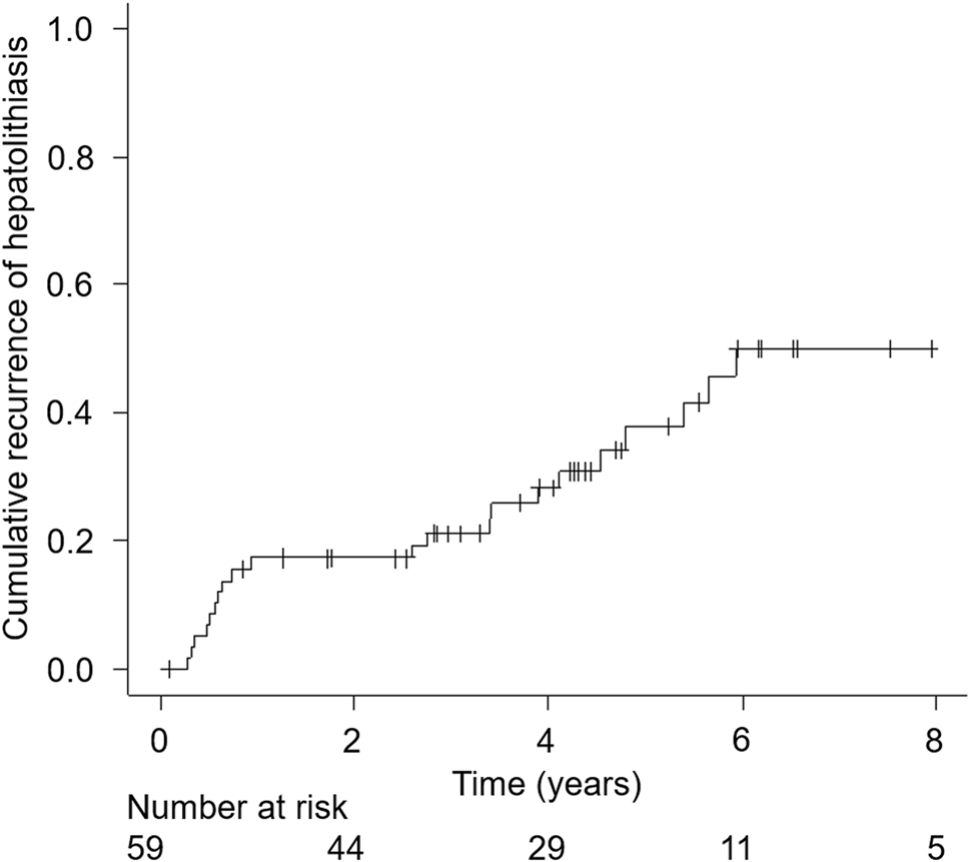
Cumulative incidence of recurrent hepatolithiasis after complete stone removal via BE-ERCP. *BE-ERCP* balloon endoscopy-assisted endoscopic retrograde cholangiopancreatography

After excluding patients with failed stone removal (*n* = 36), with biliary stent placement for stricture (*n* = 25), or who were lost to follow-up within 30 days (*n* = 11), 59 patients were eligible for long-term analyses. Cumulative incidence of recurrent hepatolithiasis was 17% in one year, 20% in three years, and 31% in five years during the median follow-up period of 3.9 years (Fig. 2).

### Comparison between past and recent HJS groups

**Fig. 3:**
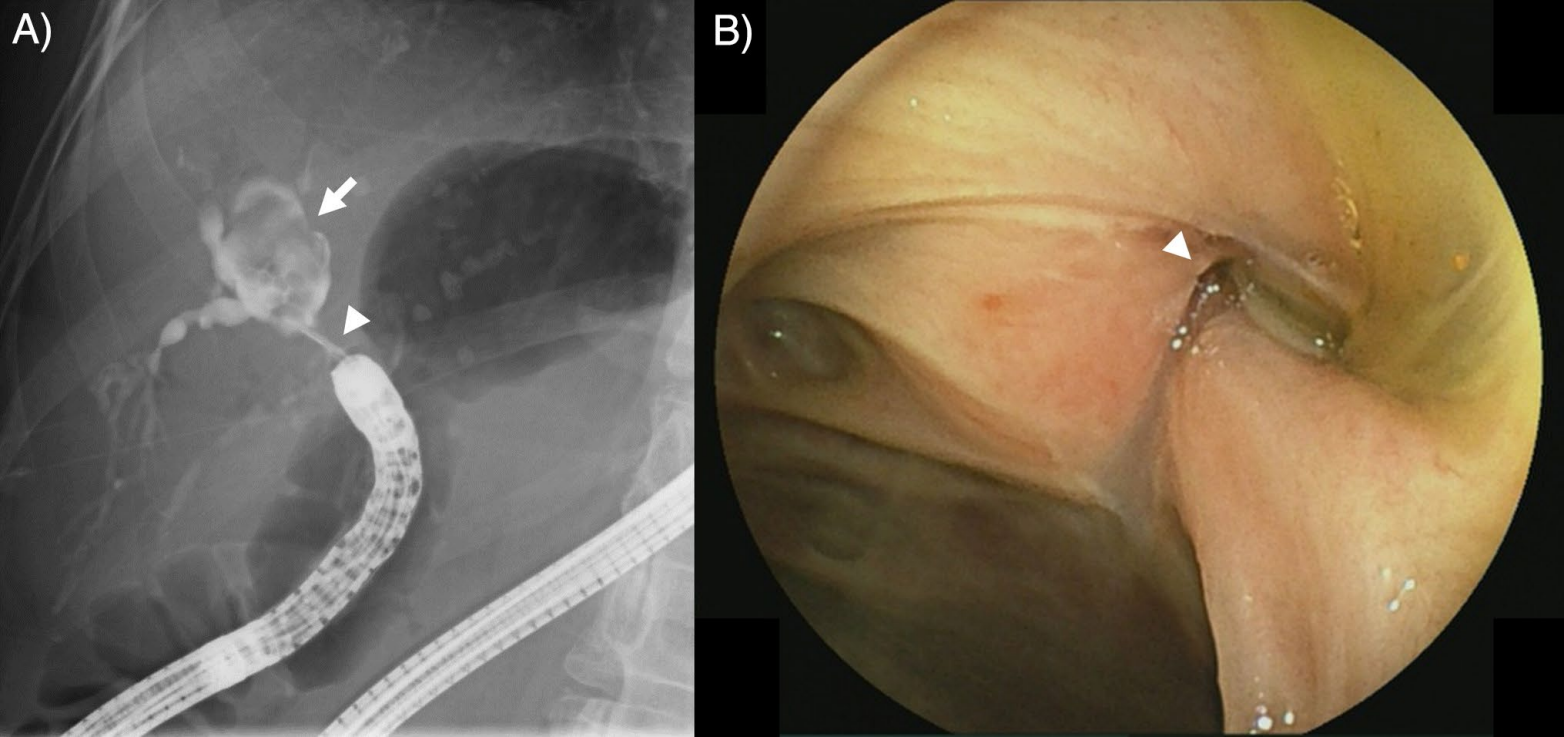
Difficult identification of the biliary orifice in a patient with previous extrahepatic bile duct resection for congenital biliary dilation. **A** Cholangiogram showed narrow right hepatic duct (white arrowhead) and large bile duct stones in a dilated peripheral bile duct (white arrow). **B** Endoscopic image of BE-ERCP. The orifice of right hepatic duct (white arrowhead) was narrowed and difficult to identify. BE-ERCP, balloon endoscopy-assisted endoscopic retrograde cholangiopancreatography

**Fig. 4:**
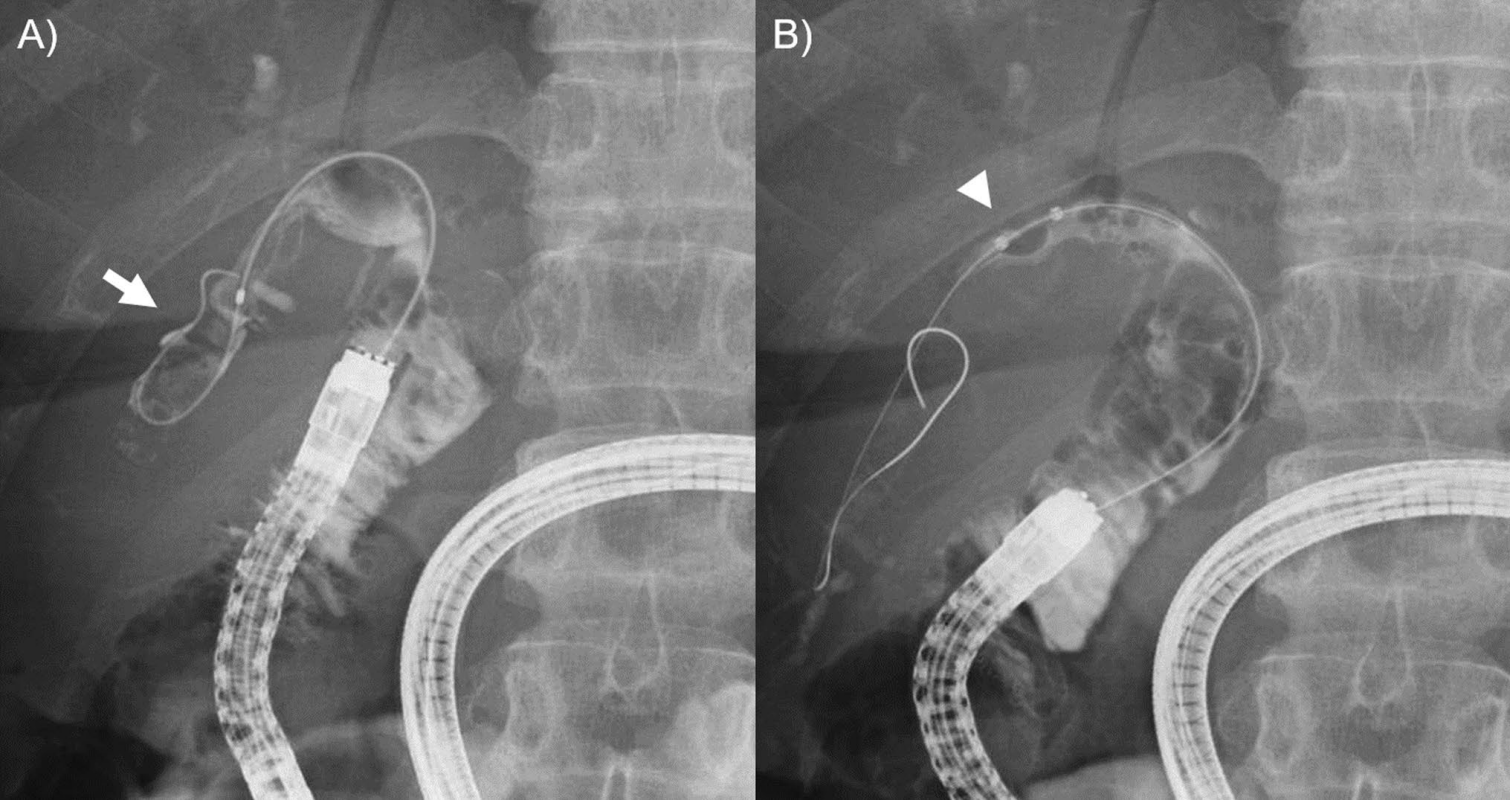
Failed device insertion into the posterior branch during BE-ERCP. **A** Cholangiogram showed hepatolithiasis (white arrow) in the peripheral bile duct of segment six. **B** A balloon catheter (white arrowhead) could not be inserted deeply due to the acute angle of bile duct and endoscope. BE-ERCP, balloon endoscopy-assisted endoscopic retrograde cholangiopancreatography

Patients in the past HJS group were younger (57 vs. 70 years old, *P* < 0.001; Table 5) and had a higher rate of congenital biliary dilation (48% vs. 16%, *P* < 0.001) and Roux-en-Y reconstruction (91% vs. 52%, *P* < 0.001). Median stone diameter and the rate of multiple stone were not different between the groups. Failed complete stone removal rates were 50% and 6.0% in the past and recent HJS groups. The rate of failed scope insertion was 20% in the past HJS group, compared with 3% in the recent HJS group. The reason for failed scope insertion was adhesion in 13% and intestinal stricture in 4.7%. Among 22% of the past HJS group with failed guidewire or device insertion to an intended bile duct, guidewire insertion failed due to the difficulty in identifying bile duct orifice in 7.8% (Fig. 3) and insertion of stone removal devices failed due to the acute angle of bile duct and endoscope in 14% (Fig. 4). The rates of total adverse events and the cumulative incidences of recurrent hepatolithiasis were similar between the groups (*P* = 0.99, and *P* = 0.75, respectively; Supplementary Fig. 2).

**Table 5:** Comparison between the past and recent HJS groups

	Patients		
Characteristic and outcomes^a^	Past HJS (*n* = 64)	Recent HJS (*n* = 67)	*P* value
Gender, male	29 (45%)	39 (58%)	0.16
Age, years	57 (40–68)	70 (57–77)	< 0.001
Primary disease			< 0.001
Malignant tumor	11 (17%)	40 (24%)	
Congenital biliary dilation	31 (48%)	11 (16%)	
Congenital biliary atresia	6 (9.4%)	0	
Other benign diseases	16 (25%)	16 (24%)	
GI reconstruction			< 0.001
Roux-en-Y	58 (91%)	35 (52%)	
Billroth-II	4 (6.2%)	32 (48%)	
Other	2 (3.1%)	0	
Biliary stricture	28 (44%)	46 (69%)	0.005
Maximum stone diameter, mm	10 (7–14)	9 (6–13)	0.44
Multiple stone^b^	37 (58%)	43 (64%)	0.48
Time from surgery to BE-ERCP, years	20 (15–31)	3 (1–5)	< 0.001
Complete stone removal via BE-ERCP	32 (50%)	63 (94%)	< 0.001
Reason for failed stone removal via BE-ERCP			
Failed scope insertion	13 (20%)	2 (3%)	
Failed guidewire or device insertion to bile duct	14 (22%)	1 (1.5%)	
Other	5 (7.8%)	1 (1.5%)	
Early adverse events	6 (9.4%)	7 (10%)	0.99

*BE-ERCP* balloon endoscopy-assisted endoscopic retrograde cholangiopancreatography, *GI* gastrointestinal, *HJS* hepaticojejunostomy

^a^Data are expressed as number (percentage) of patients within a given group or as median (interquartile range)

^b^The number of stone two or more was defined as multiple stone

## Discussion

The current study demonstrated the effectiveness and safety of BE-ERCP for hepatolithiasis in patients with HJS. Complete stone removal via BE-ERCP was achieved in 73%. The rate of early adverse events was 9.9%, but there were no severe adverse events. Multivariable logistic regression model showed the past HJS group was the only factor associated with failed stone removal via BE-ERCP.

Before introduction of BE-ERCP, PTBD has been the first-line non-surgical treatment for hepatolithiasis in patients with SAA. By using percutaneous transhepatic cholangioscopy-guided lithotomy, complete stone clearance rate was as high as 85.3%, but the major adverse event rate was 1.6% and the mortality rate was 0.8% [4]. In general, PTBD and its related procedures are associated with higher risk of early adverse events compared with endoscopic procedure [5, 19], and it developed 26% in our cohort. Meanwhile, the low rate of early adverse events in our study suggested safety of endoscopic removal of hepatolithiasis even in patients with SAA. Ishihara et al. also reported a similarly high complete stone removal rate with a low-adverse event rate in the management of hepatolithiasis via BE-ERCP [20], but evidences are still lacking to date on comparison between PTBD and BE-ERCP in this setting. Given its safety profile, BE-ERCP could be utilized as a first-line treatment for hepatolithiasis in patients with HJS, but further comparative studies are mandatory.

Our study revealed different clinical outcomes according to the timing of surgery. Contrary to the satisfactory high complete stone removal rate of 94% in the recent HJS group, it was as low as 50% in the past HJS group. There were two major reasons for technical failure in the past HJS group. One was failed scope insertion and the other was failed guidewire or device insertion to an intended bile duct. At first, 20% of patients failed scope insertion in the past HJS group. Compared with adult surgery, pediatric surgery was reported to have a higher risk of post-operative adhesive small bowel obstruction [21, 22]. Furthermore, one retrospective study reported technical difficulty of BE-ERCP in patients undergoing pediatric surgery [23]. In lines with previous studies, adhesion was the most frequent cause of failed scope insertion in the past HJS group in our study. Although CT findings might predict failed non-surgical management of adhesive small bowel obstruction [24], further investigation is needed whether failed scope insertion can be predictable by the imaging studies prior to BE-ERCP. Secondly, about 50% of patients had undergone HJS for congenital biliary dilation in the past HJS group. Hepatolithiasis after HJS in patients with congenital biliary dilation was reportedly observed in about 10% [25, 26]. Furthermore, type IV-A in Todani’s classification with dilated intrahepatic bile ducts is frequently associated with hepatolithiasis after surgery [27, 28]. Due to the narrowed orifice of dilated peripheral bile ducts, insertion of guidewire or devices for stone removal is often technically difficult in these patients.

The rate of complete stone removal raised up to 90% by rescue PTBD or EUS-HGS after failed BE-ERCP in this study. EUS-HGS is a potential salvage treatment for hepatolithiasis in patients with SAA [15, 29], and there are some case reports for successful removal of hepatolithiasis using HGS route [30]. Furthermore, direct cholangioscopy through the HGS route and subsequent electrohydraulic lithotripsy under a direct visualization could be an option for large stones [31]. However, data on the safety and effectiveness of EUS-HGS for hepatolithiasis are still limited to date, and furthermore, removal of hepatolithiasis in the right lobe is challenging through HGS route [32]. Considering the low complete stone removal rate via BE-ERCP in the past HJS group, EUS-HGS might be a good alternative first-line treatment in this cohort. However, further a large-scale study is needed to clarify the effectiveness and safety of EUS-HGS for hepatolithiasis in patients with SAA, in comparison with BE-ERCP or PTBD.

The cumulative incidence of recurrent hepatolithiasis was 17% in the first year, and it seemed to be higher compared with a previous study [20]. Complete stone removal was confirmed by cholangiography in this study, but it was sometimes difficult to distinguish remnant stone and pneumobilia. Direct cholangioscopy through an overtube was reported to be effective for confirming remnant bile duct during BE-ERCP, but this technique could be hardly available in patients with Roux-en-Y reconstruction [20]. In our study cohort, 71% of patients had Roux-en-Y reconstruction and we did not routinely perform direct cholangioscopy to confirm complete stone removal. Furthermore, 32% of our cohort was congenital biliary dilation, and these patients were likely to recur hepatolithiasis due to their morphology of the bile duct [28]. The high rate of recurrent hepatolithiasis in our study justified careful follow-up with imaging studies even after complete stone removal.

Consecutively performed BE-ERCP data and a relatively large sample size were the major strength of our study. To the best of our knowledge, the current study included the largest number of patients who underwent BE-ERCP for hepatolithiasis. Our study had several limitations. At first, the current study was a single-center retrospective design and could contain a risk of selection bias. Second, patients who underwent PTBD or surgery as a first line treatment were not included in the analyses. Third, the rate of gastrointestinal perforation was 3.1% in the current study, which was comparable to that in a large-scale prospective study [8] but higher than that in the standard ERCP. Careful periprocedure monitoring is necessary for this potentially severe adverse events in cases undergoing BE-ERCP. Fourth, technical difficulty of BE-ERCP could be different by the afferent limb length, which could be different by surgeons, institutions, countries, and time periods. The current study had a single center design and needed external validation before generalizing our results. Finally, only 59 patients were included in the long-term analyses, and this small sample size was the limitation of our study.

In conclusion, BE-ERCP was effective and safe for hepatolithiasis, but the complete stone removal rate was low in the past HJS group. Rescue EUS-HGS or PTBD was helpful to achieve completer stone removal after failed BE-ERCP. Recurrent hepatolithiasis was common and careful follow up study is needed even after complete stone removal.

### Supplementary Information

Below is the link to the electronic supplementary material.

Supplementary file1 Algorithm for stone removal via balloon endoscopy-assisted endoscopic retrograde cholangiopancreatography (TIF 121 KB)

Supplementary file2 Cumulative incidence of recurrent hepatolithiasis between the past and recent HJS groups after complete stone removal via BE-ERCP. *BE-ERCP* balloon endoscopy-assisted endoscopic retrograde cholangiopancreatography, *HJS* hepaticojejunostomy (TIF 515 KB)
