# Supplementary figures and images for: Balloon endoscopy-assisted endoscopic retrograde cholangiopancreatography for hepatolithiasis in patients with hepaticojejunostomy
Source: Surg Endosc. 2024 Mar 7;38(5):2423–32. doi: 10.1007/s00464-024-10738-6 (PMC11078785; doi:10.1007/s00464-024-10738-6)

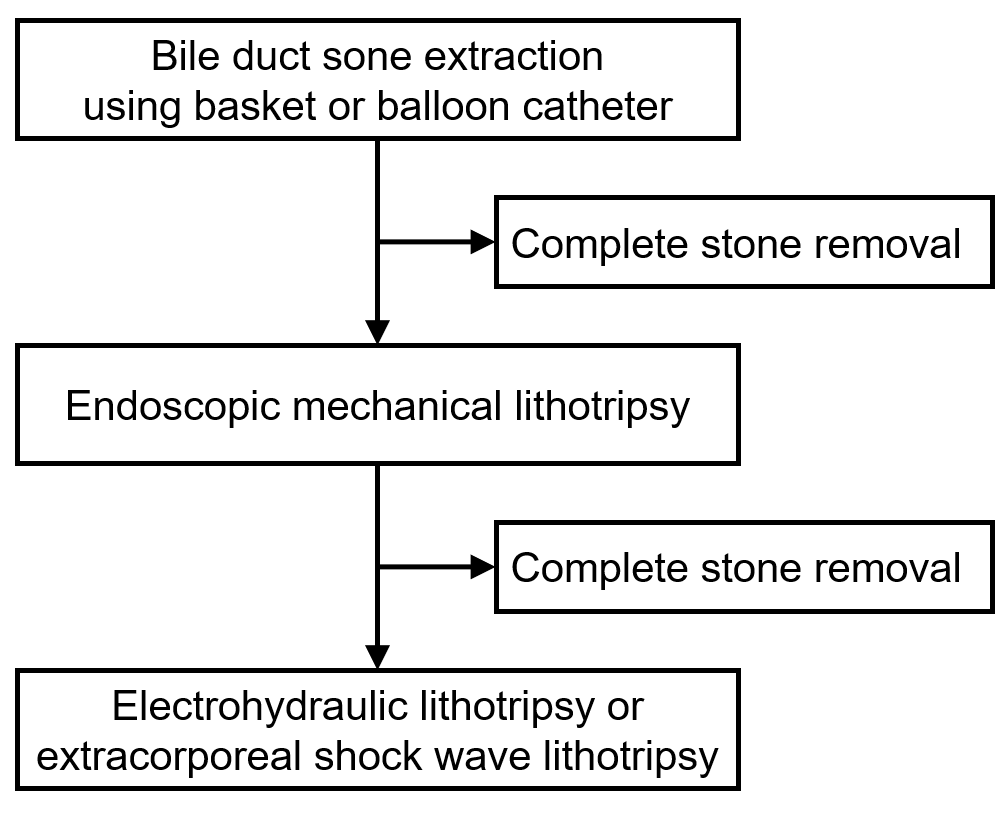

Supplement: Supplementary file 1 — Supplementary file1 Algorithm for stone removal via balloon endoscopy-assisted endoscopic retrograde cholangiopancreatography (TIF 121 KB) [file 464_2024_10738_MOESM1_ESM.tif]

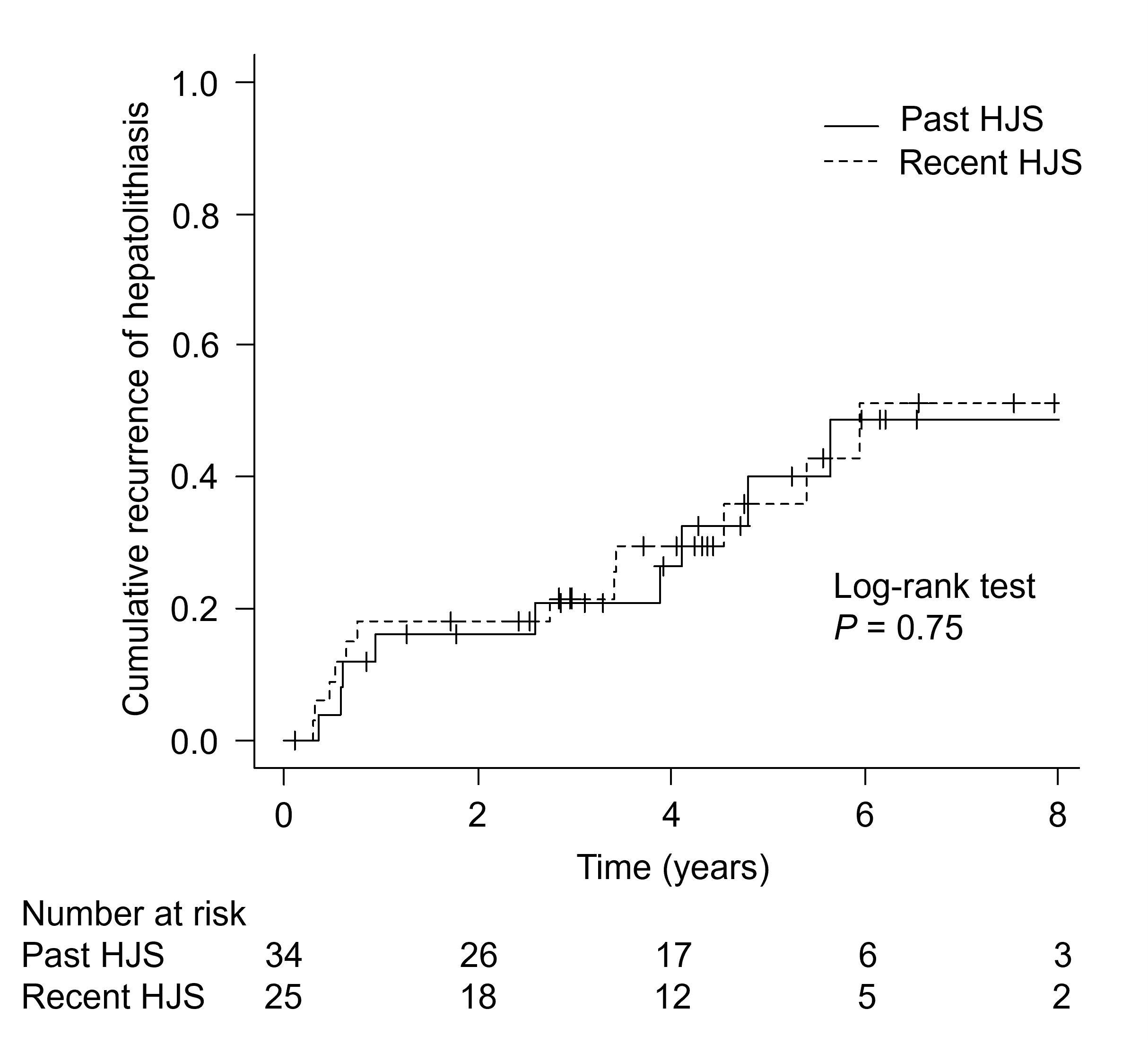

Supplement: Supplementary file 2 — Supplementary file2 Cumulative incidence of recurrent hepatolithiasis between the past and recent HJS groups after complete stone removal via BE-ERCP. BE-ERCP balloon endoscopy-assisted endoscopic retrograde cholangiopancreatography, HJS hepaticojejunostomy (TIF 515 KB) [file 464_2024_10738_MOESM2_ESM.tif]
